# The association of travel burden with prenatal care utilization, what happens after provider-selection

**DOI:** 10.1186/s12913-024-11249-9

**Published:** 2024-07-09

**Authors:** Songyuan Deng, Yuche Chen, Kevin J. Bennett

**Affiliations:** 1 University of South Carolina School of Medicine, Columbia, SC USA; 2 Department of Civil and Environmental Engineering, College of Engineering and Computing, University of South Carolina, Columbia, SC USA

**Keywords:** Prenatal care, Geographic distance, Rural, Perceived travel burden

## Abstract

**Background:**

Birthing people in the United States face numerous challenges when accessing adequate prenatal care (PNC), with transportation being a significant obstacle. Nevertheless, previous studies that relied solely on the distance to the nearest provider cannot differentiate the effects of travel burden on provider selection and care utilization. These may exaggerate the degree of inequality in access and fail to capture perceived travel burden. This study investigated whether travel distances to the initially visited provider, to the predominant PNC provider, and perceived travel burden (measured by the travel disadvantage index (TDI)) are associated with PNC utilization.

**Methods:**

A retrospective cohort of people with live births were identified from South Carolina Medicaid claims files in 2015–2018. Travel distances were calculated using Google Maps. The estimated TDI was derived from local pilot survey data. PNC utilization was measured by PNC initiation and frequency. Repeated measure logistic regression test was utilized for categorical variables and one-way repeated measures ANOVA for continuous variables. Unadjusted and adjusted ordinal logistic regressions with repeated measure were utilized to examine the association of travel burdens with PNC usage.

**Results:**

For 25,801 pregnancies among those continuously enrolled in Medicaid, birthing people traveled an average of 24.9 and 24.2 miles to their initial and predominant provider, respectively, with an average TDI of -11.4 (SD, 8.5). Of these pregnancies, 60% initiated PNC in the first trimester, with an average of 8 total visits. Compared to the specialties of initial providers, predominant providers were more likely to be OBGYN-related specialists (81.6% vs. 87.9%, *p* < .001) and midwives (3.5% vs. 4.3%, *p* < .001). Multiple regression analysis revealed that every doubling of travel distance was associated with less likelihood to initiate timely PNC (OR: 0.95, *p* < .001) and a lower visit frequency (OR: 0.85, *p* < .001), and every doubling of TDI was associated with less likelihood to initiate timely PNC (OR: 0.94, *p* = .04).

**Conclusions:**

Findings suggest that the association between travel burden and PNC utilization was statistically significant but of limited practical significance.

**Supplementary Information:**

The online version contains supplementary material available at 10.1186/s12913-024-11249-9.

## Introduction

Prenatal care (PNC) can prevent or reduce the incidence of adverse birth outcomes by providing pregnancy-related information on nutrition, fetal development, and delivery [1–3]. Adequate PNC is associated with up to a 75% reduction in maternal mortality rates [4, 5], while inadequate PNC is associated with elevated perinatal mortality rates [6]. For high-risk pregnancies, it is recommended that birthing people initiate PNC earlier and attend more PNC visits [7]. Unfortunately, approximately 1.6% of birthing people in the United States receive no prenatal care, while an additional 15.0% receive inadequate PNC [8].

Birthing people in underserved areas encounter various obstacles when accessing adequate PNC, [9] with transportation being the most significant one [10–13]. Most studies have assessed the distance from a birthing person’s residence to the nearest delivery site or PNC provider. However, this strategy raises several concerns, primarily the two-step nature of seeking perinatal care. For most patients, a physicians’ referral network provides available options for subsequent care. Among those options, patients may choose a provider considering factors such as travel distance or insurance (the first step of provider selection), and then receive care from that provider (the second step of care utilization) [14]. Travel burden may impact patients’ behavior differently in both choosing a provider and care utilization.

The second concern is that relying solely on the distance to the nearest provider may exaggerate the degree of inequality in access [15]. The two-step process, provider selection and care utilization, is dynamic but not static; experience, knowledge, and referrals from the last and current providers will contribute to the provider-selection of the next visit. Even if birthing people initially visit the nearest provider, patients also bypass these nearby providers to seek care at a more distant PNC provider due to higher medical needs, availability/accessibility of providers, or personal preferences [14, 16]. Over the course of the pregnancy, PNC providers who provide the most services to patients become the predominant PNC source, despite the distance [17].

The distances to all available PNC providers play a crucial role in initial provider selection, and influence the subsequent choice of a referral provider as well [18]. One study found that a birthing individual would typically bypass more than 90 closer providers to receive PNC from their predominant PNC provider [14]. Therefore, whether travel burden is still associated with PNC utilization remains unknown after the provider selection process, and may only impact the behavior of receiving care differently between the initial and predominant providers. This study aims to answer whether travel burden to the initial or predominant PNC provider is associated with PNC initiation and frequency.

Travel burden is a metric that has been quantified by objective metrics, including travel distance, travel time, travel cost, and availability of transportation options in previous studies [14, 19]. Subjective opinions related to transportation can also influence people’s travel behavior and their assessment of travel burdens [20, 21]. Additionally, high-risk populations perceive a greater transportation burden compared to their counterparts [22]. In these studies, the measures of perceived travel burden encompass not only transportation expenses and accessibility but also safety concerns and other barriers [20]. Even with similar travel distances and mobility options, a birthing individual with comorbidities, including but not limited to morning sickness, severe anemia, cardiac and respiratory diseases, and bone fractures, may perceive their travel burden for PNC differently from those without. Access to other general healthcare facilities, such as laboratories and pharmacies, is also crucial for medical management and thus should be included in the assessments of overall travel burden. However, no prior study has investigated the effect of a patient’s perceived travel burden on PNC utilization.

South Carolina offers a good context to explore these questions, given its higher poverty rates and uninsured rates compared to the national average [23]. Medicaid beneficiaries may also have greater travel-related needs to access PNC [24] Using South Carolina Medicaid claims data related to live births from 2015 to 2018, this study aims to investigate whether travel distances to the initially visited and the predominant PNC provider are associated with timely PNC initiation and PNC frequency, given the selection of PNC providers; and examine whether patients’ perceived travel burden is associated with these two utilization measures.

## Methods

Data (claims and enrollment) including live births covered by Medicaid in South Carolina facilities during 2015–2018 were acquired. Medicaid enrollment files supplied pertinent administrative information for all birthing people enrolled in Medicaid. The claims data furnished information regarding PNC use and associated diagnoses. Exemption was granted by the Institutional Review Board at the authors’ institution due to the secondary analysis of de-identifiable administrative data.

During 2015–2018, 108,441 live births occurred among birthing people enrolled in Medicaid in South Carolina. PNC services provided by providers were identified using Current Procedural Terminology (CPT) codes, Healthcare Common Procedure Coding System (HCPCS) codes, and ICD-9/10-CM codes. Based on the timeline of all PNC visits for a pregnancy, both the initial PNC provider and the predominant PNC provider (the provider who billed for a majority of PNC services) were identified [25]. Discontinuous enrollment in Medicaid could lead to missed visits and bias the identification of the predominant PNC provider. Therefore, only pregnancies with full coverage during the pregnancy until delivery were included in this study, resulting in a final sample of 30,020 pregnancies: 25,801 with PNC services and 4,219 without. (Appendix Figure A).

Travel burden was assessed using two measures: travel distance and perceived travel burden (also referred to as the transportation disadvantages index, TDI) [20]. Travel distance was measured as the road distance between the centroid of a patient’s area (e.g. ZCTA) to the centroid of healthcare provider’s ZCTA(?). For pregnancies with at least one PNC service and an identifiable predominant PNC provider, the actual road distance was computed from the centroid of the zip code tabulation area (ZCTA) of the birthing individual’s residence to the centroid of the provider’s ZCTA using Google Maps. If both ZCTAs were the same, the estimated radius was assigned as the average travel distance within the ZCTA, employing values from the 2010 Census ZCTA area [26] with the function: $A=\pi {r}^{2}$.

The TDI is an index that estimates an individual’s perceptions of burden when accessing necessary opportunities using various modes of transportation. The index was calculated using survey data from a representative sample of South Carolina residents in a previous study [27]. A negative TDI score indicates a higher level of perceived difficulty, correlating with greater distances traveled. The TDI utilized residents’ perceptions of the ease of travel, their capacity to travel, and their perceptions of travel safety. Individual-level travel disadvantage indices were converted into a population-weighted index at each ZCTA based on socio-demographic characteristics within the ZCTA. If a ZCTA has a high TDI, individuals residing in that area are likely to perceive greater difficulty in traveling to different places [20].

Two ordinal measures were employed to assess PNC utilization: PNC initiation for receiving the first PNC care after selecting the initial provider (the first, second or third trimesters) and PNC frequency for receiving PNC care throughout the pregnancy after selecting the predominant provider (more, adequate and less). PNC frequency was categorized into three levels: more than adequate (> 14 visits); adequate (9–14 visits); and less than adequate (1–8 visits) [28, 29].

Covariates (Appendix Table A) included socio-demographics of birthing people (age and race, dual eligibility for Medicaid and Medicare), medical needs (number of pregnancy-related conditions, number of other conditions, repeated pregnancy, age-adjusted Charlson Comorbidity Index (ACCI) [30]), specialty of the initially visited and the predominant PNC provider (specialists, midwife, nurse, primary care physician, other specialties, and organizations), and ZCTA characteristics (rurality, provider population density, uninsured rates, median income, birth rates, percentage of birthing persons with a high school degree, prevalence of birthing people using tobacco, prevalence of birthing people with obesity prior pregnancy, percentage of birthing people with less than 5 PNC visits). Rurality was defined as rural or urban [31] using 2010 rural-urban commuting area (RUCA) codes (1.0–3.0, 4.1, 5.1, 7.1, 8.1, and 10.1 as urban, and the rest as rural). To estimate the provider population density, two types of providers were used: registered PNC providers using state licensure data and identified predominant PNC providers using Medicaid claims data. The South Carolina licensure data 2013–2019 were used to estimate the annual number of active PNC providers within each ZCTA, calculated as the total number of active days divided by 365. Specialties with following abbreviations were included: OCC (Critical Care Medicine for Obstetrics & Gynecology), GO (Gynecological Oncology), OBG (Obstetrics & Gynecology), OBS (Obstetrics), GYN (Gynecology), MFM (Maternal fetal Medicine), NEO (Neo-Natal), and NPM (Neonatal-Perinatal Medicine). For each ZCTA, providers within a 24-minute driving distance were aggregated and divided by the birthing people population aged 15–50 in the corresponding year. Other ZCTA level information was acquired from the South Carolina SCAN project.

Characteristics of birthing people and providers were summarized using univariate and bivariate analysis. Comparisons were drawn between pregnancies with PNC and those without, and between initial and predominant providers [15]. Repeated measure logistic regression was utilized for categorical variables and one-way repeated measures ANOVA for continuous variables to account for those who had multiple pregnancies in the time period. Analyses were conducted using the GENMOD and MIXED procedures. Unadjusted and adjusted ordinal logistic regressions with repeated measure were employed to examine the association of travel burdens with PNC utilization. For interpretation purposes, both travel distance and TDI were subjected to a log-2 transformation in regression analysis, as the results can be explained for any doubled change in travel distance or TDI. Odds ratios with the corresponding 95% confidence interval were reported. Subgroup analysis was conducted for patients’ rural or urban residence. In approximately 3.5% of the sampled pregnancies, birthing people travelled more than 112 miles, covering almost the entire state, to obtain their PNC. Sensitivity analysis was conducted by excluding those who travelled more than 112 miles. Collinearity was checked with Variance Inflation Factor (VIF) and none of the interested independent variables had a VIF higher than 2. All analyses were performed using SAS software version 9.4 (SAS Institute Inc., Cary, NC) at the significance level of 95%.

## Results

Table 1 summarizes the characteristics of those included in the study, both with and without PNC. Out of the total sample size, 4,219 (14.1%) did not receive any PNC visits, and 5.8% were on their second pregnancy. The average age of the participants was 25 years old, with the majority being Black or White. Additionally, 9.0% of the birthing people resided in rural ZCTAs.

**Table 1:** Characteristics of all included birthing people with full medicaid enrollment in 2015–2018

Variables	All	Any PNC	No PNC (ref)
N	30,020	25,801	4,219
%	100.0	85.9	14.1
Second pregnancy (%)	6.3	6.4	5.8
Age (year)	25.3	25.0^***^	26.9
Race			
Black	47.8	49.1^***^	39.9
Unknown	5.6	4.9^***^	9.8
White	40.3	41.3^***^	34.6
non-Black racial minority	6.3	4.8^***^	15.7
Rural residency	9.0	9.3^***^	6.9
Transportation Disadvantages Index	-11.5	-11.4^*^	-11.9
Standard Deviation	8.5	8.5	8.4
Registered PNC provider density (n/100,000)	9.0	8.9	9.6
Predominant PNC provider density (n/100,000)	7.7	5.6^**^	8.3
Age adjusted Charlson comorbidity Index (%)			
Zero	91.2	92.0^***^	86.7
Mild or moderate	6.6	7.1^***^	3.8
Unknown	1.2	0.0	8.3
Severe	1.0	1.0	1.3
Pregnant-related complications (%)			
Zero	33.1	30.0^***^	52.5
Only one condition	29.3	30.2^***^	23.4
at least two conditions	37.6	39.8^***^	24.1
Other complications (%)			
Zero	58.9	56.0^***^	76.6
Only one condition	25.5	27.3^***^	14.4
at least two conditions	15.6	16.7^***^	9.0
Prenatal care initiation (%)			
The 1st trimester	N.A.	60.4	N.A.
The 2nd trimester	N.A.	30.1	N.A.
The 3rd trimester	N.A.	8.3	N.A.
Prenatal care frequency (n)		8.1	
> 14 visits (%)	N.A.	5.1	N.A.
9–14 visits (%)	N.A.	42.8	N.A.
1–8 visits (%)	N.A.	52.0	N.A.

Note: PNC: Prenatal care. ZCTA: ZIP Code Tabulation Areas. N.A.: not available

Repeated measure logistic regression test was utilised for categorical variables and one-way repeated measures ANOVA for continuous variables

Significance level: *: *p* < .05; **: *p* < .01; and ***: *p* < .001

Table 1 presents comparisons between birthing people with and without PNC visits. Compared to those without any PNC visits, those with at least one PNC visit were younger (26.9 vs. 25.0 years, *p* < .001), more likely to be White (34.6% vs. 41.3%, *p* < .001) or Black minority (39.9% vs. 49.1, *p* < .001), less likely to be a non-Black racial minority (15.7% vs. 4.8%, *p* < .001) or of unknown race (9.8% vs. 4.9%, *p* < .001), more likely to reside in a rural area (6.9% vs. 9.0%, *p* < .001), and less likely to have no pregnancy-related complications (52.4% vs. 30.0%, *p* < .001) or no other complications (76.6% vs. 56.0%, *p* < .001). There was a significant but slight difference in TDI scores, -11.4 and − 11.9 (standard deviation, 8.5 and 8.4, respectively, *p* = .04), between those with and without any PNC, respectively.

Regarding provider population density, birthing people who received any PNC showed no significant difference in the densities of registered PNC providers (8.9 vs. 9.6, *p* = .14) but significantly lower densities of identified predominant PNC providers (7.6 vs. 8.3, *p* < .01), compared to those without any PNC visit. Only 8.3% of birthing people without any PNC visit had an unknown ACCI (Table 1).

Among pregnancies where birthing people had any PNC, 60.4% initiated PNC in the first trimester. The average number of PNC visits was 8.1 and birthing people received fewer than 9 PNC visits for 52% of included pregnancies (Table 1).

Table 2 summaries the characteristics of both initial and predominant providers. Pregnant people travelled a longer distance to initial providers than to predominant providers (24.9 vs. 24.2 miles, *p* < .001). A change in specialty occurred from the initial PNC provider to the predominant PNC provider; birthing people were more likely to initiate PNC with primary care physicians (9.4% vs. 4.5%, *p* < .001), organizations (2.7% vs. 0.9%, *p* < .001) and providers with other specialties (0.8% vs. 0.3%, *p* < .001) and then shift to an obstetric-gynecologist (81.6% vs. 87.9%, *p* < .001) or a midwife (3.5% vs. 4.3%, *p* < .001) for most of their PNC.

**Table 2:** Characteristics of providers visited by birthing people with any prenatal care, 2015–2018

Variables	Initial provider	Predominant provider
Distance from patients’ residence (miles)^*^	24.9	24.2^***^
Specialty (%)		
Specialist	81.6	87.9^***^
midwife	3.5	4.3^***^
nurse	2.0	2.1
Primary Care Physician	9.4	4.5^***^
Others	0.8	0.3^***^
FQHC/DHEC/RHC	2.7	0.9^***^

*: *N* = 25,801. Distance was calculated from resident ZCTA to the PNC provider ZCTA, centroid to centroid by Google Map if two ZCTAs not the same. Otherwise, the average distance within a ZCTA was the calculated radius from 2010 Census ZCTA area with the function:$A=\pi {r}^{2}$

Initial providers provide the PNC initiation services. Predominant providers provide the most services for all PNC during a pregnancy

Significance level: *: *p* < .05; **: *p* < .01; and ***: *p* < .001

Table 3 presents all odds ratios (ORs) and 95% confidence intervals from ordinal logistic regressions using repeated measures. A doubled travel distance was associated with delayed initiation and lower frequency in both the unadjusted (OR: 0.98, 95% CI: 0.96-1.00, *p* = .04; OR: 0.97, 95% CI: 0.95–0.99, *p* = .009; initiation and frequency, respectively) and adjusted models (OR: 0.95, 95% CI: 0.93–0.97, *p* < .001; OR: 0.95, 95% CI: 0.93–0.97, *p* < .001; initiation and frequency, respectively). A higher perceived travel burden, defined as a doubled TDI, was associated with delayed PNC initiation in both unadjusted (OR: 0.94, 95% CI: 0.89–0.99, *p* = .02) and adjusted (OR: 0.94, 95% CI: 0.88-1.00, *p* = .04) models. A higher perceived travel burden was not significantly associated with PNC frequency in both models.

**Table 3:** Odds ratio and 95% confidence intervals from regression analysis for prenatal care use, 2015–2018

Measures	PNC initiation			PNC frequency		
Model 1, unadjusted model	OR	Lower	Upper	OR	Lower	Upper
travel distance	0.98^*^	0.96	1.00	0.97^**^	0.95	0.99
TDI	0.94^*^	0.89	0.99	0.97	0.93	1.01
Model 2, adjusted model						
travel distance	0.95^***^	0.93	0.97	0.95^***^	0.93	0.97
TDI	0.94^*^	0.88	1.00	0.99	0.95	1.03

Note: PNC: Prenatal care. TDI: Transportation Disadvantages Index

Travel distance and TDI were taken logarithm base 2 transformation

Unadjusted and adjusted ordinal logistic regressions with repeated measure were employed. Covariates, including socio-demographics of birthing people, specialties of providers, and ZCTA characteristics, were controlled for in the adjusted regression

Significance level: *: *p* < .05; **: *p* < .01; and ***: *p* < .001

Table 4 summarizes the results of subgroup analysis by rurality. For rural populations, a doubled travel distance was associated only with delayed initiation (OR 0.86, 95% CI: 0.79–0.93, *p* < .001), and TDI was not associated with either outcome. On the contrary, for urban populations travel distance did not differ from the entire study population, yet TDI was associated with both outcomes (OR: 0.91, 95% CI: 0.83–0.90, *p* = .04; OR: 0.90, 95% CI: 0.82–0.98, *p* = .02; initiation and frequency, respectively).

**Table 4:** Odds ratio and 95% confidence intervals from regression analysis for prenatal care use, subgroup analysis by patients’ residence, 2015–2018

Measures	PNC initiation			PNC frequency		
Rural (*n* = 2,407)	OR	Lower	Upper	OR	Lower	Upper
travel distance	0.86^***^	0.79	0.93	0.95	0.88	1.02
TDI	0.96	0.90	1.02	1.01	0.97	1.05
Urban (*n* = 23,394)						
travel distance	0.95^***^	0.93	0.98	0.95^***^	0.93	0.98
TDI	0.91^*^	0.83	0.99	0.90^*^	0.82	0.98

Note: PNC: Prenatal care. TDI: Transportation Disadvantages Index

Travel distance and TDI were taken logarithm base 2 transformation

Adjusted ordinal logistic regressions with repeated measure were employed. Covariates, including socio-demographics of birthing people, specialties of providers, and ZCTA characteristics, were controlled for in the adjusted regression

Significance level: *: *p* < .05; **: *p* < .01; and ***: *p* < .001

Travel pattern was plotted (figure not provided) and it was identified that some birthing people travelled almost across the whole state, covering more than 112 miles. Due to the limited detection of telehealth, a sensitivity analysis was conducted by excluding those who travelled more than 112 miles. The sample size for PNC initiation was reduced from 25,801 to 24,889 (excluding 912 with more than 112 miles to the initial provider) and for PNC frequency, it was reduced from 25,801 to 24,996 (excluding 805 with more than 112 miles to the predominant provider). The results were consistent with the prior model results, except for slight changes in coefficients.

## Discussion

This study found that the negative effect sizes of travel burdens on PNC utilization were statistically significant but practically small after the provider-selection process, among South Carolina Medicaid enrolled birthing people. The probabilities for earlier PNC initiation and frequency would decrease slightly if travel distance doubled, and the probabilities for earlier PNC initiation would decrease slightly if TDI doubled.

This study is one in a series focusing on the association between travel burden and PNC utilization. The travel burden played a more significant role in provider-selection than in receiving care. A previous study found that travel distances to all available predominant PNC providers played a significant role in provider selection. Birthing people were 14 times more likely to choose a nearer provider (within 26 miles) than those located 26–81 miles away, and 14 ~ 16 times more likely to avoid providers located further away (beyond 81 miles) [18]. Given the selected PNC provider, however, this study found a small effect size on the association between travel distance and receiving PNC services. A birthing individual in this study would only be 5% less likely to initiate PNC in an earlier trimester if the travel distance to the first visited PNC provider was doubled, compared to another birthing individual when other covariates were controlled for. Similarly, a birthing individual would be only 5% less likely to have a higher level of PNC frequency if the travel distance to the predominant PNC provider doubled, compared to another birthing individual when other covariates were controlled for. This finding indicates that resources allocated to address travel burden during receiving care would not be efficient without considering provider-selection.

To address these issues of travel burden on PNC utilization, providers and policymakers should consider evidence during both provider-selection and receiving care. The results of this study suggest that for birthing people with varying travel burdens, if they visited the same provider, there would be only a minor difference in receiving PNC. The substantial differences in the effect sizes of travel burden highlight the importance of travel distance in the provider-selection process. Therefore, policy implications should consider the provider-selection process as a major factor in PNC. Building a referral network can be one solution; peer referral networks, in particular, could play a significant role in provider-selection, [14, 32] as evidenced by a previous study that found the number of connections with other peers was a significant predictor of being selected by patients [18].

Another implication is the impact of recent closure of obstetric units [33–35] on the provider-selection process. Those closures have already added to, and will continue to add, more travel burden to affected birthing people. This study provides evidence that future studies should investigate how that closure impact birthing people’s provider-selection process rather than directly examining PNC utilizations.

To authors’ knowledge, this is the first study examining the association between travel burden to the visited providers and subsequent PNC utilization. Unlike prior studies that used travel distance to the nearest available provider to examine the impact on PNC utilization, this study applied travel distance to both the initially visited and predominant PNC providers. The rationale behind this choice is that using the travel distance to the nearest available but not necessarily visited provider would overstate the access disparity between different groups with different access to PNC [15]. Consequently, the results of this study cannot be directly compared to those of previous studies.

The inclusion of the concept of perceived travel burden represents an innovative aspect of this study, as its comprehensive exploration has been relatively lacking in prior literature. Prior research has primarily focused on objective transportation metrics and their impacts on healthcare services, such as travel distance or time to healthcare facilities, and the frequency of public transit service. However, these objective transportation metrics may not accurately capture individuals’ realized ability to travel, and their perception of travel difficulties based on their socioeconomic status. For instance, a person with a lower income may perceive transportation costs differently compared to those with higher incomes. Apart from the direct impacts of perceived travel burden on healthcare facilities, indirect effects are also important through nutrition intake and physical exercise. Nutrition education, micro-nutrient supplementation and access to fresh food are promising factors that could prevent adverse birth outcomes and enhance fetal health [36]. Furthermore, physical activity has been identified as a protective factor for adverse maternal and birth outcomes [37, 38]. However, many communities are not safe for residents to travel without a vehicle [39, 40]. Therefore, limited access to nutrient and fresh food and safety concerns for physical exercise may increase the prevalence of pregnant comorbidities, leading to increased PNC needs.

Thus, in this study, we have adopted a perceived travel difficulty index and investigate its effects on PNC utilization. This travel difficulty index includes measures of travel difficulty for both private and public transportation travel. For each travel mode, it integrated aspects of opportunity accessibility, safety concerns, cost, and abilities, through both the direct and indirect pathways, to assess travelers’ objective feelings of travel difficulties based on their social-demographic status. This is the first study investigating the association between perceived travel burden and PNC utilization.

Our results indicate a significant disparity in the perception of travel difficulty between patients who received PNC visits and those who did not. Specifically, patients who did not attend PNC visits reported encountering significantly greater travel difficulties compared to those who did. Our results also reveal that after accounting for chronic comorbidities and pregnancy-related complications, the travel disadvantage index exhibited a connection with slightly delayed PNC initiation. However, no observed correlation was found between the travel disadvantage index and the frequency of PNC visits, taking into consideration the healthcare provider that was visited. This finding aligns with the conceptual process during pregnancy, where birthing people who perceive a high travel burden may delay initiating PNC but ultimately overcome it due to the recognized benefits for both maternal and neonatal health.

The different nature of objective travel distance and subjective TDI was also evident in our subgroup analysis. For rural subjects, only objective travel distance was associated with late initiation of PNC; for the urban subjects, while both measures were barriers for PNC utilization, TDI presented a larger effect size than travel distance.

The study is subject to several limitations. First, it is important to note that the calculation of perceived travel burden is based on findings from an initial pilot survey characterized by a limited sample size. To enhance the robustness of our analysis and findings, it is worth noting that the research team is currently conducting a follow-up survey with a significantly larger sample size. The expected increase in sample size is anticipated to contribute to more robust analyses and conclusive outcomes. Ideally, the implementation of regular surveys over a specific period would be optimal, as this would continually update our understanding of the perceived travel burden among the local populace. Finally, an overall travel burden metric that integrates both objective and subjective measures would be valuable to explore.

Using data before 2014, a previous study reported that approximately 60% of American birthing people experienced a change in insurance status, transitioning from one insurance to another and potentially being uninsured for at least one month during pregnancy [41]. The total number of PNC visits is crucial for determining both PNC initiation and PNC frequency. Without complete visit information, the estimated PNC initiation and frequency could be biased. To avoid this bias, this study only included pregnancies with continuous Medicaid enrollment. However, by doing so, the study sample does not fully represent all South Carolina Medicaid beneficiaries, nor does it reflect others without Medicaid. The likelihood of timely PNC initiation in the first trimester for this study was 60.4%, which was lower than the 72.0% for the South Carolina general public and 68.1% for Americans enrolled in Medicaid in 2016 [8]. This lower initiation rate could be partly attributed to the representativeness of the sample. While the generalizability was limited by the sample characteristics, additional research should be conducted using claims from different payers and for other geographic locations.

Telehealth may introduce bias to these estimates. Some birthing people sought PNC at a greater distance from their residential ZCTAs yet had a delivery site that was in or nearer to the residential ZCTAs. These PNC encounters may have been due to travel or the use of telehealth. In 2011, the absolute rates of telehealth utilization were between 0.09% among Medicaid beneficiaries [42]. South Carolina initiated a demonstration project for obstetric-gynecologic telehealth in July 2014 [43]. However, this study confirmed only one tele-PNC visit for one live birth using the HCPCS modifiers (“GT”), and thus, it was unable to confirm or deny if these were telehealth visits. The impact of telehealth on these estimates remains unknown. To mitigate this impact, the sensitivity analysis excluded those who traveled more than 112 miles, and the main results remained consistent.

This study assigned an estimated radius to approximately 16% of pregnancies with any PNC, representing the travel distance where the residential ZCTA of birthing individuals matched that of PNC providers’ ZCTA. Computing this radius relied on two assumptions. Firstly, it assumed that the ZCTA is nearly round. Second, it presumed that the provider is positioned at the center of this round, with all patients situated on its edge. Consequently, this approach led to an underestimated variation in travel distances for this population, and thus, the results of travel distances were underestimated. Future studies that utilize actual addresses of patients and providers can circumvent this limitation.

In studies that examine travel burdens on healthcare utilization, selection bias is a concern. Patients who travel a long distance to visit their providers may not be sensitive to travel burdens. Our measure of TDI showed that perceived travel burden was almost the same for patients who travelled more than 24 miles or 24 miles or less. (data not shown) That result suggests the possible selection bias was not evident in the current study. Furthermore, the results of the sensitivity analysis showed that the main results remained consistent, after excluding those who travelled more than 112 miles.

In conclusion, this study found that the travel burden, measured by travel distance to visited providers and the perceived travel difficulty index, was statistically associated with PNC utilization in South Carolina birthing people with full enrollment during their pregnancy. However, the effect sizes were practically small for receiving care after provider-selection. Further studies are necessary to validate this conclusion, and priority should be given to exploring the association between travel burden and the provider-selection process. Policymakers should concentrate on the role of travel burden in the provider-selection process, such as providers’ referral network, to address the issue of PNC access rather than addressing it after provider-selection.

### Electronic supplementary material

Below is the link to the electronic supplementary material.

Supplementary Material 1

Supplementary Material 2

## Data Availability

The South Carolina Medicaid data that support the findings of this study are available from South Carolina Revenue and Fiscal Affairs Office, but restrictions apply to the availability of these data, which were used under license for the current study, and so are not publicly available. The primary survey data for constructing travel disadvantage index is owned by Yuche Chen. It is available upon request to the corresponding author. Software programs are available upon request to the corresponding author through email: songyuan@email.sc.edu.
