# Supplementary material for: The association of travel burden with prenatal care utilization, what happens after provider-selection
Source: BMC Health Serv Res. 2024 Jul 9;24:781. doi: 10.1186/s12913-024-11249-9 (PMC11234759; doi:10.1186/s12913-024-11249-9)
Supplement: Supplementary file 1 — Supplementary Material 1 [file 12913_2024_11249_MOESM1_ESM.docx]

Appendix Table A. Covariate list

| Variable Name | Type | Description |
| --- | --- | --- |
| **Individual characteristics** |  |  |
| Second pregnancy | Binary | Whether this is the second pregnancy for a woman |
| Age | Continuous | Age at delivery (year) |
| Race |  |  |
| White (reference group) | Binary | White or not |
| Black | Binary | African American or not |
| Other | Binary | Known race but not White and African American |
| Unknown | Binary | Unknown race |
| Transportation Disadvantages Index | Continuous | Annual average transportation disadvantages index for all live births |
| ZCTA registered PNC provider density | Continuous | Number of registered PNC providers within 30 minutes driving time per 100,000 ZCTA women aged 15-50 |
| ZCTA predominant PNC provider density | Continuous | Number of predominant PNC providers within 30 minutes driving time per 100,000 ZCTA women aged 15-50 |
| Age adjusted Charlson comorbidity Index |  |  |
| Zero (reference group) | Binary | Age adjusted Charlson comorbidity Index = 0 |
| Mild or moderate | Binary | Age adjusted Charlson comorbidity Index = 1, 2, 3, 4 |
| Unknown | Binary | Missing Age adjusted Charlson comorbidity Index |
| Severe | Binary | Age adjusted Charlson comorbidity Index > 4 |
| Pregnant-related complications^1^ |  |  |
| Zero (reference group) | Binary | No pregnant related complication |
| Only one condition | Binary | Only one pregnant related complication |
| at least two conditions | Binary | At least two pregnant related complications |
| Other complications^2^ |  |  |
| Zero (reference group) | Binary | No other complication |
| Only one condition | Binary | Only one other complication |
| at least two conditions | Binary | At least two other complications |
| **PNC provider** |  |  |
| Rural residency | Binary | Whether this provider located in a rural area |
| Distance | Continuous | Miles from mom resident ZCTA centroid to practice ZCTA centroid |
| Specialty | Categorical | OBGYN specialists, midwives, nurse practitioners, primary care physicians, others, and organizations (FQHC/DHEC/RHC) |
| ZCTA characteristics |  |  |
| Uninsurance rates | Continuous | The annual percentage of residents without any healthcare insurance |
| Median income | Continuous | The annual family median income |
| Birth rates | Continuous | The annual number of live births per thousand of population |
| Educational achievement | Continuous | The annual percentage of mothers with a high school degree |
| Tobacco use | Continuous | The annual prevalence of pregnant women using tobacco |
| Obesity prior pregnancy | Continuous | The annual prevalence of pregnant women with obesity prior pregnancy |
| PNC use | Continuous | The annual percentage of pregnant women with less than 5 PNC visits |

1: Pregnant-related complications include pre-existing hypertension; gestational proteinuria or hypertension; pre-eclampsia; eclampsia; other hypertension; diabetes mellitus in pregnancy, childbirth, and the puerperium; malnutrition in pregnancy, childbirth and the puerperium; excessive weight gain/obesity in pregnancy; low weight gain in pregnancy; pregnancy care for patient with recurrent pregnancy loss; excessive vomiting in pregnancy; multiple gestation; complications specific to multiple gestation; maternal care for mal-presentation of fetus; maternal care for known or suspected fetal abnormality and damage; maternal care for disproportion; maternal care for abnormality of pelvic organs; placental disorders; placenta Previa; premature separation of placenta; polyhydramnios; premature rupture of membranes; other disorders of amniotic fluid and membranes; uterine size date discrepancy; antepartum hemorrhage; hemorrhage in early pregnancy; drug use; tobacco use disorder; and other mental disorders and diseases of the nervous system.

2: Other complications include anemia complicating pregnancy, childbirth and the puerperium; venous complications and hemorrhoids in pregnancy; other diseases of the blood and blood-forming organs and certain disorders involving the immune mechanism; other endocrine, nutritional and metabolic diseases; abnormal glucose; liver and biliary tract disorders in pregnancy, childbirth and the puerperium; infections of genitourinary tract in pregnancy; maternal infectious and parasitic diseases; diseases of the circulatory system; diseases of the respiratory system; diseases of the digestive system; diseases of the skin and subcutaneous tissue; and other specified diseases and conditions.
