# Supplementary figures and images for: The association of travel burden with prenatal care utilization, what happens after provider-selection
Source: BMC Health Serv Res. 2024 Jul 9;24:781. doi: 10.1186/s12913-024-11249-9 (PMC11234759; doi:10.1186/s12913-024-11249-9)

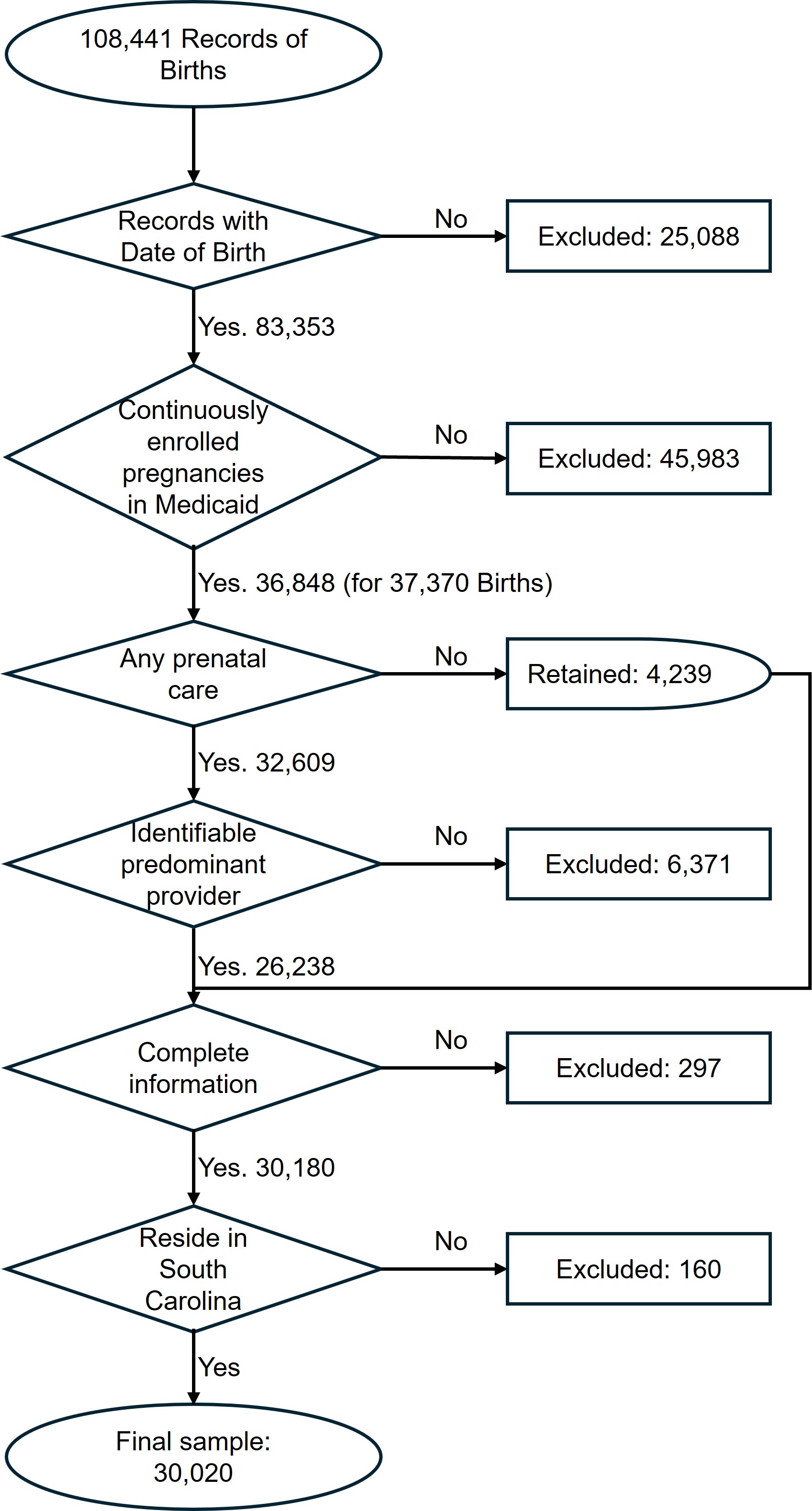

Supplement: Supplementary file 2 — Supplementary Material 2 [file 12913_2024_11249_MOESM2_ESM.jpg]
